# HIF-inducible miR-191 promotes migration in breast cancer through complex regulation of TGFβ-signaling in hypoxic microenvironment.

**DOI:** 10.1038/srep09650

**Published:** 2015-04-13

**Authors:** Neha Nagpal, Hafiz M. Ahmad, Shibu Chameettachal, Durai Sundar, Sourabh Ghosh, Ritu Kulshreshtha

**Affiliations:** 1 Department of Biochemical Engineering and Biotechnology, Indian Institute of Technology Delhi, India-110016; 2 School of Life Sciences, Jawaharlal Nehru University, New Delhi, India- 110067; 3 Department of Textile Technology, Indian Institute of Technology Delhi, India-110016

## Abstract

The molecular mechanisms of hypoxia induced breast cell migration remain incompletely understood. Our results show that hypoxia through hypoxia-inducible factor (HIF) brings about a time-dependent increase in the level of an oncogenic microRNA, miR-191 in various breast cancer cell lines. miR-191 enhances breast cancer aggressiveness by promoting cell proliferation, migration and survival under hypoxia. We further established that miR-191 is a critical regulator of transforming growth factor beta (TGFβ)-signaling and promotes cell migration by inducing TGFβ2 expression under hypoxia through direct binding and indirectly by regulating levels of a RNA binding protein, human antigen R (HuR). The levels of several TGFβ pathway genes (like VEGFA, SMAD3, CTGF and BMP4) were found to be higher in miR-191 overexpressing cells. Lastly, anti-miR-191 treatment given to breast tumor spheroids led to drastic reduction in spheroid tumor volume. This stands as a first report of identification of a microRNA mediator that links hypoxia and the TGFβ signaling pathways, both of which are involved in regulation of breast cancer metastasis. Together, our results show a critical role of miR-191 in hypoxia-induced cancer progression and suggest that miR-191 inhibition may offer a novel therapy for hypoxic breast tumors.

Hypoxia (pO_2_, <5–10 mm Hg) is a regular feature of breast tumor microenvironment and has often been associated with poor prognosis^1^. It affects a variety of tumor properties such as proliferation, migration, invasion, epithelial to mesenchymal transition, angiogenesis, vascularization and apoptosis^2^. Besides, it also leads to therapeutic failure by promoting resistance to ionizing radiation and various chemotherapeutic drugs^3^,^4^. Thus, understanding of hypoxia signaling has been an active area of research. Specific hypoxia regulated genes such as *HIF* (hypoxia inducible factor), *VEGFA* (vascular endothelial growth factor A) and *CA9* (carbonic anhydrase 9) have been identified as promising targets for cancer therapy or as diagnostic/prognostic markers in clinical research^5^,^6^,^7^.

A major advance in the understanding of hypoxia signaling was made with our discovery that hypoxia, apart from regulating protein coding genes, also regulates a class of small, endogenous, non-coding RNAs called microRNAs (miRNAs)^8^. Until now, more than 2500 of miRNAs have been discovered in humans, however, functions are known of very few of them. miRNAs play an important role in disease physiology and pathogenesis through fine tuning of gene expression^9^,^10^. They are conventionally known to bind to the 3′UTR of target genes and bring about their degradation or translational repression depending on the degree of complementarity with the target site^11^. However, recent studies suggest that miRNA binding sites are not limited to the 3′UTR but may involve the coding, 5′UTR or promoter regions of the target genes^12^,^13^. Additional major revelation has been that miRNAs may induce the target gene expression as well, that may include mechanisms such as their interplay with RNA binding proteins, interaction with promoter elements (TATA box motifs) and 5′UTR of the target genes^14^,^15^,^16^,^17^,^18^. These findings suggest the versatility of miRNA mode of action and cellular functions.

The repertoire of hypoxia regulated miRNAs (HRMs) may vary depending upon the cellular or physiological context, however, specific HRMs consistently show hypoxia inducibility in diverse contexts^8^,^19^. The most prominent and well studied among these is miR-210 that was found to be robustly induced by hypoxia across various cell types^20^. The hypoxic regulation of HRMs has been found to be HIF dependent, though it is speculated that other hypoxia relevant transcription factors such as p53 and nuclear factor-Kappa B may be involved^21^. Specific HRMs such as miR-210, miR-373, miR-155 and miR-10b have been shown to regulate diverse functions such as angiogenesis, apoptosis, DNA repair, migration and cell proliferation to fine tune the cellular response to hypoxia^22^,^23^,^24^,^25^. This corroborates the crucial role of HRMs in mediating cellular adaptation to hypoxia in tumor cells. However, the functions of most of the HRMs remain unknown.

Here, we have focused our work on miRNA, miR-191, that was found to be hypoxia inducible in breast cancer^26^. Higher levels of miR-191 have been reported before in several cancers including breast cancer and it has been shown to function as an oncogenic miRNA in some of these (breast, hepatic, colon and gastric cancers)^27^. miR-191 was identified as an important player in estrogen signaling that controls various oncogenic properties in breast cancer, though in a context dependent manner^26^,^28^. However, its association or functions under hypoxia have not been investigated yet.

Our results here identify miR-191 as a HIF regulated miRNA that promotes proliferation, migration and therapeutic resistance under hypoxia. miR-191 overexpression under hypoxia leads to induction of TGFβ pathway. We further show that miR-191 induces TGFβ2 transcript by direct binding and by regulation of levels of RNA binding protein, HuR (Human antigen R) specifically under hypoxia. miR-191 mediated TGFβ2 induction promotes breast cancer cell migration. Overall, considering strong impact of miR-191 on breast cancer biology, it emerges as a potential therapeutic target in the treatment of breast cancer.

## Results

### miR-191 is hypoxia inducible in a HIF dependent manner

**Figure 1:**
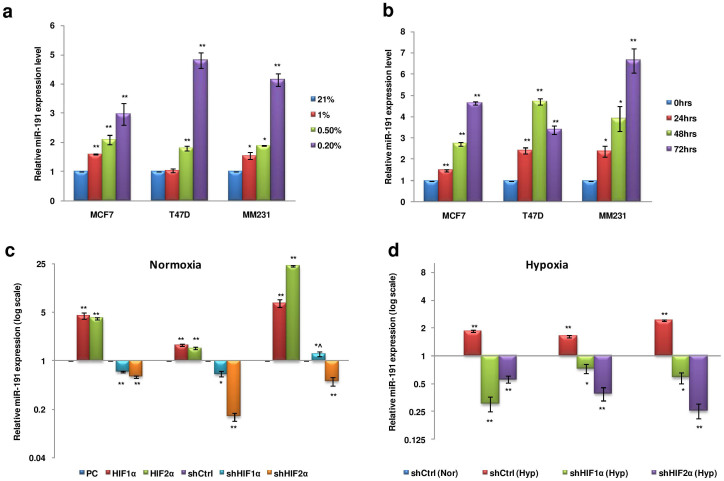
Transcriptional regulation of miR-191 in hypoxic microenvironment. (a). Stem loop qRT-PCR data showing relative level of miR-191 in breast cancer cell lines (MCF7, T47D, & MM231) exposed to different pO_2_ (0.2%, 0.5%, 1% & 21%) for 48 hrs. (b). Stem loop qRT-PCR data showing time dependent induction of miR-191 levels in response to 0.2% hypoxia treatment in MCF7, T47D, & MM231 cells. (c, d). Effect of HIF overexpression (HIF-1α or HIF-2α) or inhibition (shHIF1α or shHIF2α) in normoxic (c) or hypoxic (d) conditions on the levels of miR-191 in a panel of breast cancer cell lines (MCF7, T47D, & MM231). The graphical data points represent mean ± S.D of at least three independent experiments. (*P < 0.05, **P < 0.01, *^P > 0.05 < 0.1). Error bars denote ± SD.

Tumor microenvironment plays a major role in breast cancer tumorigenesis^29^. However, recent studies have expanded the influence of tumor microenvironment to migration/invasion and polarity of cancer cells that may involve the role of miRNAs^30^. We recently found that the stress prevalent in tumor microenvironment has an inducing effect on mature miR-191 levels^26^. We have now extended our study to further evaluate the effect of hypoxic stress on mature-miR-191 levels. We took a panel of breast cancer cell lines (MCF7, T47D, MM231) and exposed them to different O_2_ concentrations for 48 hrs. We found that severe hypoxic microenvironment (pO_2_-0.2%) has a maximum inducing effect on mature miR-191 levels as opposed to moderate hypoxia (1%) (Figure 1a). Thus, all the subsequent experiments were performed at 0.2% pO_2_. A time dependent induction in mature miR-191 levels (4–7 fold induction) in response to hypoxic stress was seen, suggesting it to be chronic in nature (Figure 1b).

We next wanted to study the mechanism behind miR-191 induction under hypoxia. Since HIFs are master regulators of hypoxia response, we thus sought to study their involvement in miR-191 regulation^31^. We transiently modulated the level of HIFs (HIF-1α & HIF-2α) and scored for the effect on miR-191 levels. Interestingly, overexpression of both the HIFs led to strong induction in miR-191 levels while considerable reduction in miR-191 levels was observed with the silencing of the HIFs using specific shRNAs under normoxia (Figure 1c). HIF silencing under hypoxia using specific shRNAs led to a drastic decrease in miR-191 levels suggesting that hypoxia mediated induction of miR-191 is HIF dependent (Figure 1d). The validity of hypoxic microenvironment or functionality of the HIF overexpression or silencing constructs was tested by evaluating their effect on HRE (hypoxia/HIF response element) luciferase construct of a hypoxic marker gene, erythropoietin (EPO-HRE) (Supplementary data 2a, b). Altogether, these results suggested that hypoxia mediated strong induction in miR-191 levels is HIF-dependent.

**Figure 2:**
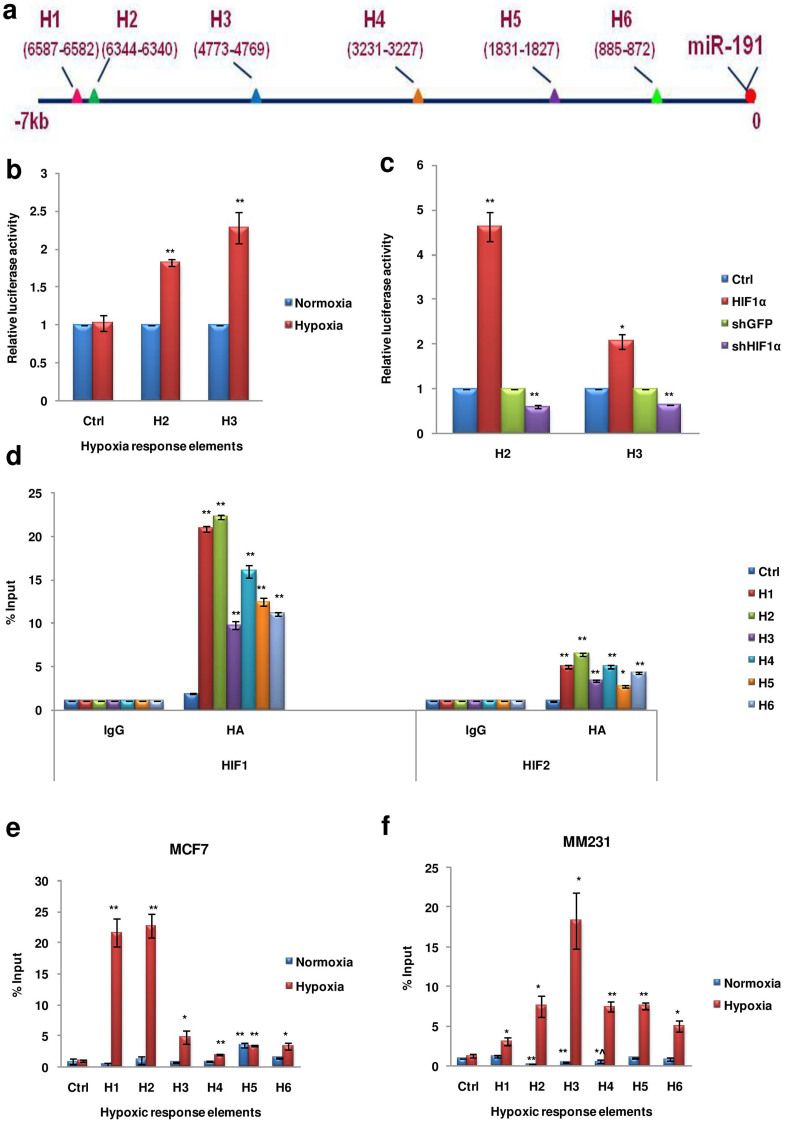
Transcriptional regulation of miR-191 is HIF dependent. (a). Picture Diagram showing six potential hypoxia response elements (HRE) upstream of pre-miR-191 sequence. (b, c). The functionality of the putative hypoxia response elements (H2 & H3) was confirmed through dual luciferase assay in response to hypoxia (b) or HIF stimulation/inhibition (c). Graph represents relative luciferase activity. (d). MCF7 cells were transfected with HA tagged constructs of HIF 1/2-α and exposed to hypoxia for 24 hr. CHIP assay was performed using antibodies against IgG or HA tag to validate the binding of HIF 1/2-α on the putative HREs in the promoter of miR-191. Bar graph represents fold enrichment of bound chromatin compared with input chromatin. (e, f). Cells were exposed to normoxia or hypoxia for 24 hr. CHIP assay was performed using antibodies against IgG or HIF-α. Bar graph represents fold enrichment of bound chromatin compared with input chromatin in MCF7 (e) and MM231 (f) cell lines. Ctrl refers to region lacking in HIF-binding sites. The graphical data points in b-f represent mean ± S.D of at least three independent experiments. (*P < 0.05, **P < 0.01, *^P > 0.05 < 0.1). Error bars denote ± SD.

Next, to identify if HIFs are involved in the direct regulation of miR-191, we looked for the presence of potential HREs in the region upstream of the precursor miR-191 sequence. The HRE of all the hypoxia regulated genes contain the core sequence (A/G)CGTG, mostly ACGTG^32^. We thus searched for this core sequence (ACGTG) in the region upto 7 kb upstream of precursor miR-191 and identified 6 potential regulatory sites (HI-H6) (Figure 2a). Two of the putative promoter elements (H2 & H3) were then cloned in a luciferase reporter vector (pGL3-tk-Luc) and their functionality was confirmed through luciferase activity in response to hypoxia or HIF-stimulation/inhibition (Figure 2b, c). Enhanced luciferase activity was observed on HIF/hypoxia stimulation suggesting a direct role of these HREs in hypoxia/HIF induced miR-191 levels. To further confirm HIF binding at these sites, we performed chromatin immunoprecipitation (CHIP) assay by transiently overexpressing HIFs in the hypoxic microenvironment. Interestingly, HRE binding was more enriched in both HIF-1α (~20 fold higher) & HIF-2α (~3–5 fold higher) fractions as compared to the control (Rabbit-IgG) antibody (Figure 1d). These results strongly demonstrated that in response to hypoxia, HIFs are recruited to the HRE sites in miR-191 promoter to induce miR-191 transcript levels. Notably, HIF-1α showed more robust effect than HIF-2α. Next, to confirm the dynamic recruitment of HIF-1α to these HREs upstream of miR-191(H1-H6) following hypoxic exposure, CHIP assay was performed in MCF7 cells with antibody specific to HIF-1α/Rabbit IgG and cells at 21% oxygen (normoxia) were taken as control (Supplementary data 2c). In agreement with the previous results, HIF-1α showed strong binding to the HREs (specifically H1-H3 showed ≥5 fold increase) in hypoxic cells as compared to the normoxic cells (Figure 2e). Similar results were obtained when CHIP assay was done in MM231 cells (using antibody specific to HIF-1α/rabbit IgG) further confirming the binding of HIF-1α on putative miR-191 promoter HREs (Figure 2f). Thus, miR-191 induction under hypoxia is mediated by recruitment of HIF to the HRE sites present in its promoter.

### Hypoxia inducible miR-191 promotes breast tumor aggressiveness

**Figure 3:**
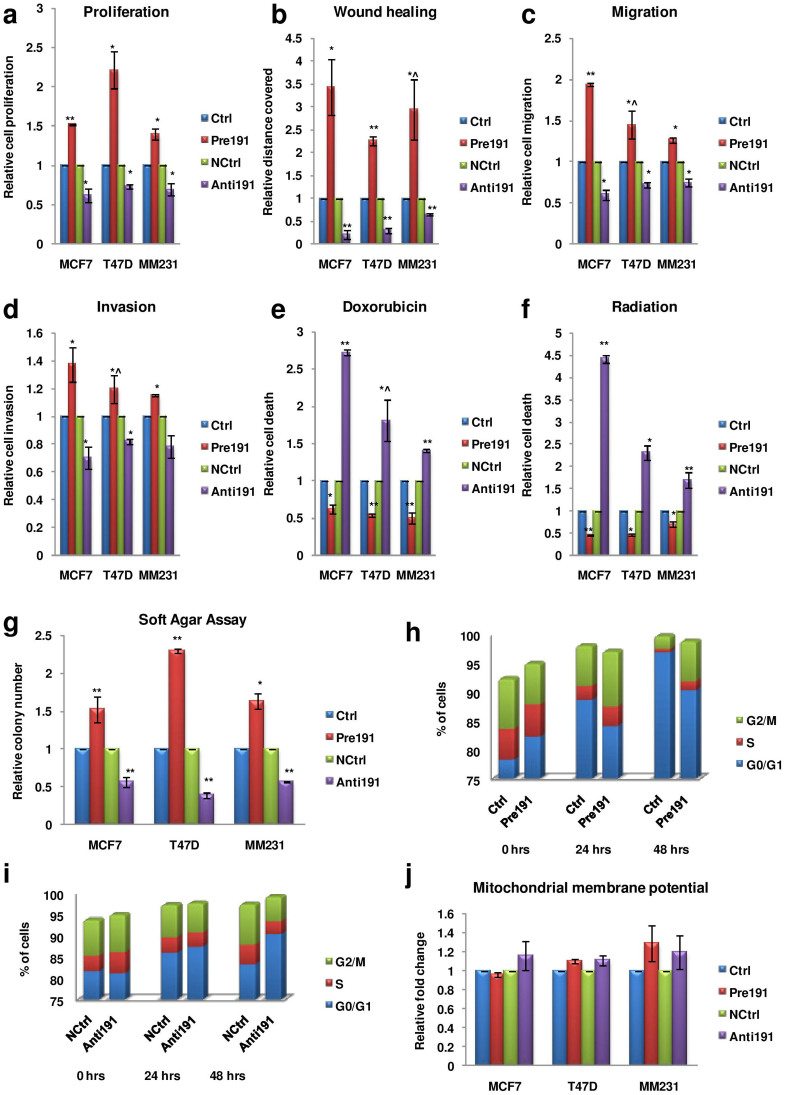
miR-191 promotes breast cancer aggressiveness. (a–f). Levels of miR-191 were modulated (overexpressed-Pre191 or inhibited-Anti191) in hypoxic microenvironment in a panel of breast cancer cell lines (MCF7, T47D & MM231) and various cellular effects were studied. Bar graphs represent effect of miR-191 level modulation on (a) Cell proliferation using MTT assay (b) Migration using wound healing assay (c) Migration using Boyden chamber assay (d) Invasion using Boyden chamber assay (e) Cell death using Trypan blue assay in reponse to chemotherapeutic drug (doxorubicin) or (f) Raditaion treatment.(gamma rays-2 Gray) (g). Soft Agar assay of cells transiently transfected with pre or anti-miR-191 with respective controls (n = 3) and cells were allowed to grow in hypoxic microenvironment. (h, i). Cell cycle analysis was performed with serum starvation synchronized MCF7 cells followed by differential modulation of miR-191 levels and exposure to hypoxic microenvironment. FACS analysis shows reduction in cells in G0/G1 phase while increase in cells in G2/M phase on miR-191 overexpression (h) while inhibition of miR-191 shows opposite (i). (j). The cells were transfected with miR-191 mimic or inhibitor with respective controls and exposed to hypoxia for 24 hrs. Cells were then stained with JC-1 for 15 min at RT. Mitochondrial membrane potential was measured as a ratio of red (JC-1 aggregates) to green (monomers) fluorescence intensity. Bar graph represents relative fold change as compared to control. The graphical data points represent mean ± S.D of at least three independent experiments. (*P < 0.05, **P < 0.01). Error bars denote ± SD.

Hypoxia has been strongly associated with breast tumor aggressiveness since it promotes cellular proliferation, migration, invasion and drug resistance properties^33^. We thus checked whether hypoxia inducible miR-191 acts as a mediator of the same. Since we wanted to identify miR-191 specific effects with respect to hypoxia, thus all experiments have been carried out under hypoxia and comparisons have been made between cells transfected with control oligos versus pre/anti-miR-191(Supplementary data 3a, b). Overall, we found that miR-191 overexpression promoted cell proliferation, migration, invasion (modest but statistically significant) and resistance to chemotherapeutic drug (doxorubicin) or radiation mediated death while miR-191 downregulation using anti-miR-191 oligos showed the opposite effect as compared to the control in all the three cell lines tested (Figure 3a–f).

Anchorage independent growth is another hallmark of cellular tumorigenesis. The soft agar assay was thus performed to explore the role of miR-191 in encouraging the anchorage independent growth under hypoxia. Interestingly, considerably higher number of colonies were observed when cells with increased level of miR-191 were exposed to hypoxia and allowed to grow on semi-solid media while miR-191 inhibition showed the opposite (Figure 3g).

To investigate the potential mechanism of miR-191 mediated cell proliferation, we next performed cell cycle analysis using flow cytometry. MCF7 cells were arrested in G0/G1 phase by serum starvation and these arrested cells were washed with 20% serum and then transfected with miR-191 oligos [sense/antisense or controls (ctrls)] and exposed to hypoxic microenvironment in complete media. Cells were collected at various time points and stained with PI (propidium iodide) for FACS (fluorescence-activated cell sorting) analysis. Notably, miR-191 overexpressing cells showed reduction in cells in G0/G1 phase as compared to respective controls. Concomitantly there was increase in cells in G2/M phase on miR-191 overexpression. In contrast, inhibiting miR-191 under hypoxia showed higher cells in G0/G1 phase and fewer G2/M phase cells as compared to the control suggesting that miR-191 inhibition under hypoxia causes cell cycle arrest (Figure 3h, i).

Since miR-191 affects the cells in apoptotic peak (G0/G1) we sought to know whether it is due to its effects on mitochondrial functioning. JC-1 (a mitochondrial membrane potential sensitive dye) was used for this purpose. Cells with differential miR-191 levels were exposed to hypoxia for 24 hrs and stained with JC-1. The ratio of red (JC-1 aggregates) to green (monomers) fluorescence intensity was determined for each sample as a representative of ΔΨm. However, no change was observed in the JC-1 aggregates/monomers in the cells with differential miR-191 levels suggesting that mitochondrial functioning remains unaffected due to miR-191 levels under hypoxia (Figure 3j).

### miR-191 induces TGFβ-signaling under hypoxia

**Figure 4:**
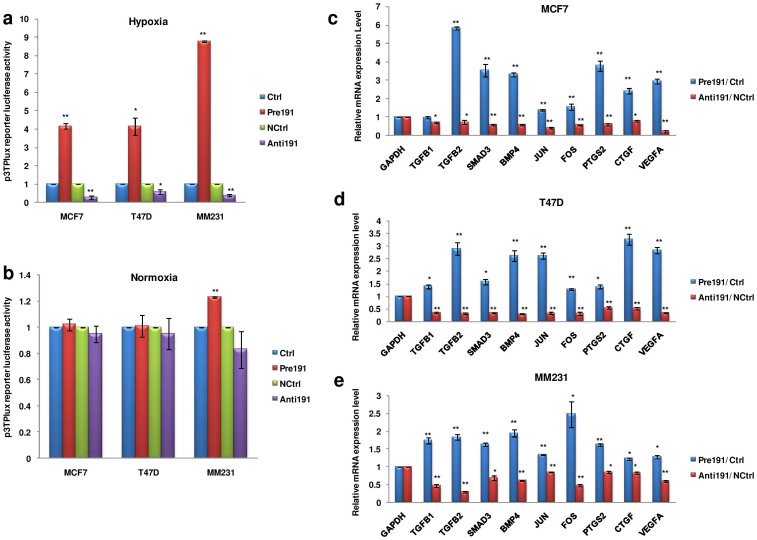
miR-191 promotes TGFβ-signaling in hypoxic microenvironment. (a, b). Bar graph represents relative luciferase activity of p3TP-Lux reporter plasmid on overexpression/inhibition of miR-191 under hypoxic (a) and normoxic conditions (b). (c–e). qRT-PCR data showing expression level of genes belonging to TGFβ-pathway in response to miR-191 overexpression/inhibition in hypoxic microenvironment in a panel of breast cancer cell lines- MCF7 (c), T47D (d), MM231 (e). The graphical data points in a-e represent mean ± S.D of at least three independent experiments. (*P < 0.05, **P < 0.01). Error bars denote ± SD.

The TGFβ-signaling pathway is known to control breast cancer proliferation, metastasis and invasion^34^,^35^. Further, a recent study in hepatocellular carcinoma suggested a link between miR-191 and TGFβ pathway based on gene expression data. We thus investigated the involvement of TGFβ-signaling in miR-191 mediated increased breast cancer migration under hypoxia. To begin with, we first determined the effect of miR-191 on TGFβ-signaling by using a 3TP-Lux reporter plasmid (possessing TGFβ-responsive elements)^36^. Substantial increase in luciferase activity was observed when miR-191 mimic was cotransfected along with the reporter plasmid in hypoxic microenvironment while inhibition of miR-191 led to strong reduction in luciferase activity in the three breast cancer cell lines (Figure 4a). Under normoxic conditions no change in luciferase activity was observed suggesting that miR-191 mediated induction of TGFβ signaling is hypoxia specific (Figure 4b). We next checked the level of TGFβ pathway genes known to be involved in breast cancer metastasis^37^,^38^,^39^,^40^,^41^,^42^. Interestingly, we saw increase in the levels of *TGFβ2, SMAD3* (SMAD family member 3), *BMP4* (bone morphogenetic protein 4), *JUN, FOS, PTGS2* (prostaglandin-endoperoxide Synthase 2), *CTGF* (connective tissue growth factor), & *VEGFA* transcripts in response to miR-191 overexpression and their downregulation on miR-191 inhibition under hypoxia in the three cell lines (Figure 4c–e). Overall, these results suggest existence of an association between miR-191 and TGFβ-signaling in the hypoxic microenvironment.

### TGFβ2: A direct miR-191 target in hypoxic microenvironment

**Figure 5:**
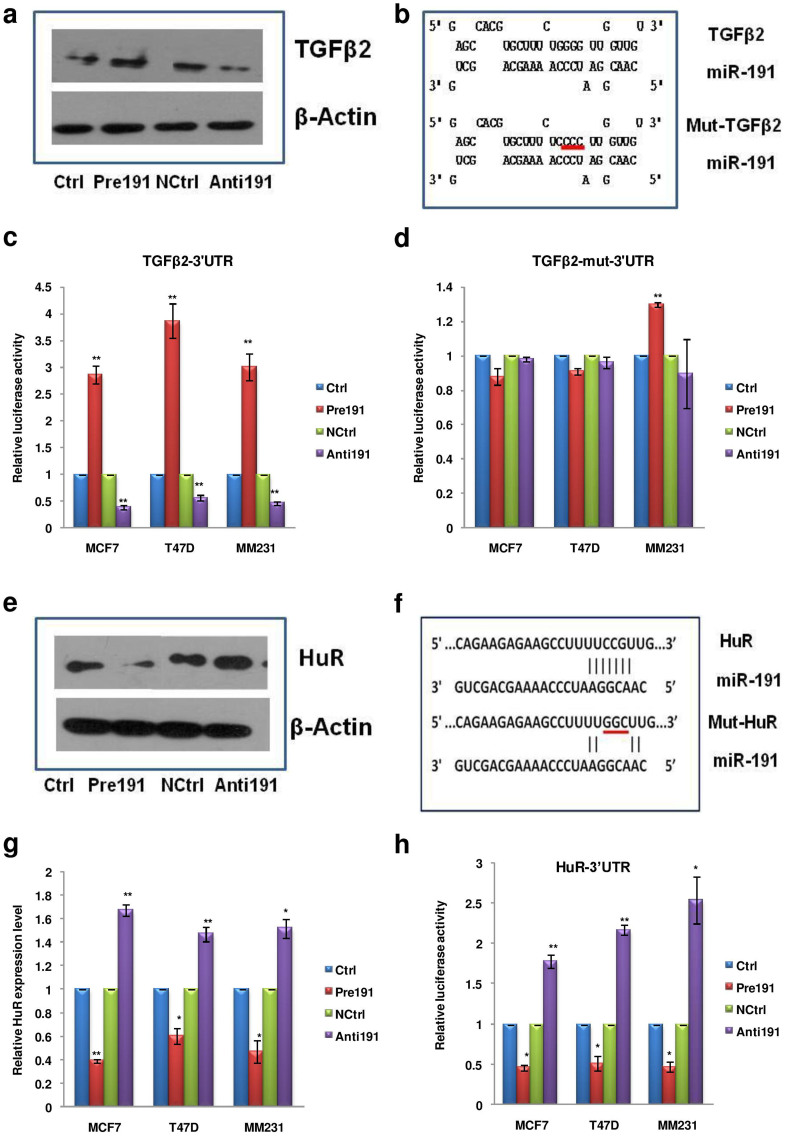
TGFβ2 & HuR: bonafide targets of miR-191 in hypoxic microenvironment. (a). The MCF7 cells with differential level of miR-191 were exposed to hypoxic microenvironment and analyzed for expression of TGFβ2 protein by western blotting. (b). Diagram showing wild type/mutated miR-191 binding site in TGFβ2-3′UTR. (c, d). Graph showing 3′UTR luciferase activity of TGFβ2-3′UTR luciferase constructs bearing wild type (c) or mutated (d) miR-191 binding site, in response to differential expression of miR-191 in hypoxic microenvironment. (e). The MCF7 cells with differential level of miR-191 were exposed to hypoxic microenvironment and analyzed for expression of HuR protein by western blotting. (f). Diagram showing miR-191 binding site (HuR B1, wild type as well as mutated) in the 3′UTR of HuR that were subsequently cloned and scored for luciferase activity. (g). qRT-PCR data showing that miR-191 overexpression (Pre191) leads to targeted downregulation of HuR at the transcript level while its inhibition (Anti191) leads to the opposite. (h). Bar graph showing relative luciferase activity of HuR-3′UTR luciferase construct in response to miR-191 level modulation. The graphical data points in c, d, g, h represent mean ± S.D of at least three independent experiments. (*P < 0.05, **P < 0.01). Error bars denote ± SD.

We were interested to know the reason behind the upregulation of various members of the TGFβ pathway. Since TGFβ2 showed maximal induction and is also an upstream molecule in the TGFβ pathway we sought to see if it is a direct target of miR-191. We first determined the effect of miR-191 on TGFβ2 protein level. The MCF7 cells with differential level of miR-191 were exposed to hypoxic microenvironment (pO_2_-0.2%, 24 hrs) and analyzed for the expression of TGFβ2 protein by western blotting and immunofluorescence using an antibody specific for TGFβ2. Interestingly, overexpression of miR-191 resulted in increased levels of TGFβ2 protein while its inhibition led to the opposite (Figure 5a, Supplementary data 4a, c).

To confirm if TGFβ2 is a bonafide target of miR-191, we sought help from PicTar5, miRWalk & RNAhybrid (binding prediction with consideration of G:U base pairing in both UTR & CDS region) target prediction programs and found a potential miR-191 binding site in TGFβ2 3′UTR^43^,^44^,^45^ (Figure 5b). We cloned this putative site downstream of a luciferase reporter vector and quantitated luciferase activity on cotransfecting the Pre191/Anti191/Control oligos along with these reporters constructs in the three cell lines. The cells were exposed to hypoxic microenvironment for 24 hours post transfection. Interestingly, the luciferase assay results were in agreement with the qRT-PCR data showing that miR-191 directly targets TGFβ2 (Figure 5c). The results were further validated by mutating (four point mutations) the miR-191 binding sites in TGFβ2 3′UTR. The relative luciferase activity of the reporter constructs containing TGFβ2 –mut-3′UTR was found to be unaltered by the presence of either of miR-191 mimic or inhibitor (Figure 5d) in contrast to the wild type TGFβ2-3′UTR reporter construct. It confirmed that miR-191 binds to the 3′UTR of TGFβ2 (at position 5756–5784) and brings about its induction in hypoxia. Notably, under normoxia, miR-191 was unable to modulate TGFβ2 levels suggesting hypoxia specificity of this effect (Supplementary data 4e–g).

### miR-191 mediated downregulation of HuR in hypoxia leads to induction of TGFβ2

RNA-binding proteins, such as HuR, play a very important role in hypoxia signaling by regulating the levels of various transcripts at post-transcriptional level by binding to U- or AU rich sequences in their 3′UTRs^46^,^47^. Using target prediction programs (TargetScan, PicTar5, miRanda, miRWalk) we found that HuR is a predicted target of miR-191 and there are two possible miR-191 binding sites in the 3′UTR of HuR transcript^11^,^43^,^44^ (Supplementary data 5a). Using qRT-PCR, luciferase reporter activity assays & western blotting, we validated that overexpression of miR-191 suppresses the expression of HuR in hypoxic microenvironment while its inhibition led to the opposite (Figure 5e–h, Supplementary data 4b). To further confirm HuR as a direct target of miR-191, the two miR-191 binding sites (HuR B1 and HuR B2) in HuR 3′UTR were individually or together cloned (HuR B1&2) and 3′UTR luciferase activity was scored (Supplementary data 5a, b). It was found that miR-191 mediated HuR downregulation was mainly through the HuR B1 while HuR B2 seemed largely unresponsive. The HuR B1 site was mutated for further confirmation and as expected, miR-191 overexpression led to decrease in luciferase activity with wild type HuR B1 construct while no effect was observed when the mutated HuR B1 was used (Supplementary data 5c). Altogether, this confirmed that miR-191 directly targets HuR by binding to its 3′UTR at position 4027–4033. Similar to TGFβ2, miR-191 mediated regulation of HuR was not seen under normoxia suggesting this effect to be hypoxia-specific (Supplementary data 4e–g).

**Figure 6:**
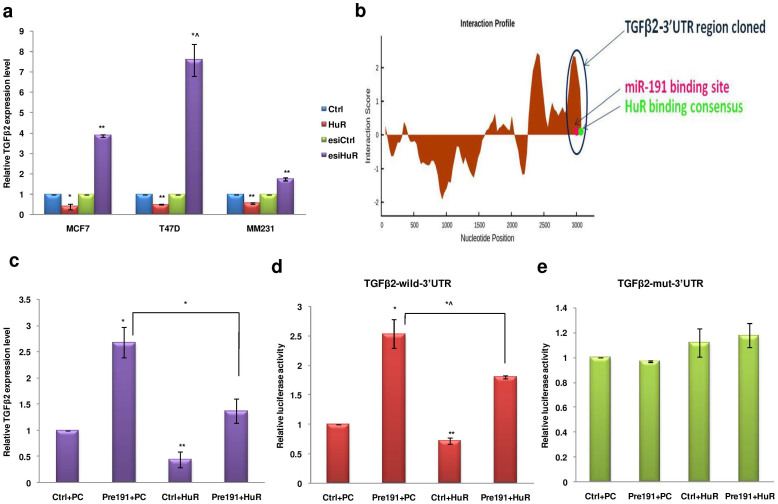
Targeted downregulation of HuR plays an important role in miR-191 mediated TGFβ2 induction in hypoxic microenvironment. (a). Effect of HuR on the level of TGFβ2 transcript in hypoxic microenvironment. Overexpression of HuR led to reduction in level of TGFβ2 mRNA while Silencing the HuR (esiHuR) led to significant induction in level of TGFβ2 mRNA. (b). Interaction profile of HuR protein with TGFβ2 3′UTR. The cloned 3′UTR region of TGFβ2 encompassing the binding sites of miR-191 and HuR is highlighted. The region was considered for determining the combined effect of both miR-191 and HuR in the regulation of TGFβ2. (c). qRT-PCR data showing regulation of TGFβ2 levels in MCF7 cells in response to overexpression of miR-191 or HuR or both. (d, e). Graph showing luciferase activity of TGFβ2-wild type (d) or mutated 3′UTR (e) in response to overexpression of miR-191 or HuR or both in MCF7 cells. Results show that miR-191 mediated induction of TGFβ2 was partially reduced in presence of HuR overexpression. The graphical data points in a & c-e represent mean ± S.D of at least three independent experiments. (*P < 0.05, **P < 0.01, *^P > 0.05 < 0.1). Error bars denote ± SD.

The mechanism of miRNA mediated induction in target transcript levels has been linked to its interplay with the RNA binding proteins^15^. Given the links between hypoxia, HuR and miR-191 and between miR-191 and TGFβ2, we investigated whether HuR is involved in the regulation of TGFβ2 levels. Strikingly, HuR overexpression led to reduction in the level of TGFβ2 mRNA and silencing HuR (esiHuR) led to considerable induction in the level of TGFβ2 mRNA in hypoxic microenvironment (Figure 6a, Supplementary data 3c–e). However, under normoxia, no or reduced effect of HuR modulation on TGFβ2 levels was observed (Supplementary data 4d).

We next did *in-silico* analysis for the presence of HuR binding consensus (NNUUNNUUU) in TGFβ2-3′UTR and identified a total of 19 HuR binding sites (HuR consensus sequences; NNUUNNUUU) (Supplementary data 6)^48^. We calculated the interaction score for HuR binding to the 3′UTR region of TGFβ2 transcript using catRAPID software and found that HuR binding sites close to miR-191 binding site showed very high interaction score (Figure 6b)^49^. Since miR-191 and HuR both were showing contrasting effects on TGFβ2 levels and were found to have binding sites in proximity, we checked whether HuR binding affects miR-191-TGFβ2 transcript interaction. To experimentally determine this we sought for the effect of miR-191 on TGFβ2 level in the presence of HuR in hypoxic MCF7 cells using qRT-PCR (Figure 6c). We found that miR-191 mediated induction of TGFβ2 was partially reduced in presence of HuR overexpression. Further, luciferase assay using TGFβ2-UTR (wild type as well as mutated) also confirmed the same (Figure 6d, e). This suggests that miR-191 mediated downregulation of HuR is important to bring about full TGFβ2 induction.

### TGFβ2 is a critical mediator of miR-191 induced breast cancer migration

**Figure 7:**
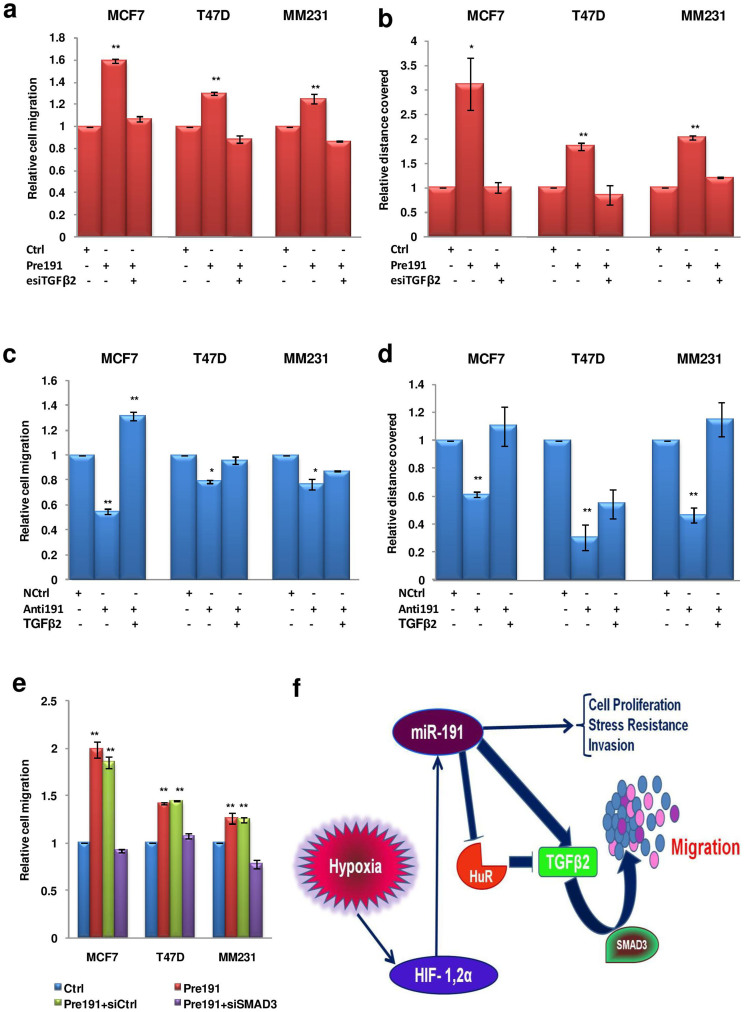
TGFβ2 is a critical mediator of miR-191 induced breast cancer migration under hypoxia. (a, b). Effect of miR-191 and TGFβ2 on migration under hypoxia. (a, b) The cells were transfected with control miRNA or miR-191mimic along with TGFβ2 silencing using esiTGFβ2 and migration was quantitated using Boyden chamber assay (a) or wound healing assay (b). The results from the three cell lines show that miR-191 mediated increase in migration is abolished with the TGFβ2 silencing. (c, d) The cells were transfected with control miRNA or anti-miR-191 along with TGFβ2 overexpression and migration was quantitated using Boyden chamber assay (c) or wound healing assay (d). The results show that the decrease in migration by inhibition of miR-191 was reduced when TGFβ2 was coexpressed. (e). miR-191 induced migration is SMAD3 dependent as reduction in migration was observed on miR-191 overexpression along with the inhibition of SMAD3. (f). A proposed model detailing hypoxic regulation of miR-191 along with its functional impact on HIF & TGFβ-pathways and further implication in regulation of migration of hypoxic breast cancer. The graphical data points in a-e represent mean ± S.D of at least three independent experiments. (*P < 0.05, **P < 0.01). Error bars denote ± SD.

To further gain insight into association of miR-191 and TGFβ-pathway in the promotion of breast cancer migration, we examined whether the silencing/restoration of TGFβ2 affects miR-191 induced cell migration. We performed transwell migration and wound healing assay with cells co-transfected with an esiRNA (esiTGFβ2) for TGFβ2 along with miR-191 mimic. Notably, the increase in migration (~2 fold) seen with miR-191 mimic alone was abolished when miR-191 mimic was used along with esiTGFβ2 (Figure 7a, b, Supplementary data 3f, h). Similarly, while anti-miR-191 led to decrease in migration, forced expression of TGFβ2 along with anti-miR-191 oligos led to restoration of migration (Figure 7c, d, Supplementary data 3g, h). Thus, results obtained from all the three cell lines suggest that miR-191 mediated breast cancer migration under hypoxia is TGFβ2 dependent.

TGFβ2 pathway may function in a SMAD dependent or independent manner^50^. Thus, to determine whether miR-191:TGFβ2 induced breast cancer migration is SMAD3 dependent, we silenced SMAD3 by using SMAD3-specific siRNA and analyzed the effect on miR-191 induced breast cancer migration. Enhanced cell migration was observed when miR-191 mimic was used alone or along with siCtrl, however the effect was abolished when miR-191 mimic was used along with siSMAD3 (Figure 7e, Supplementary data 3i). Taken together, these findings suggest that hypoxia induced miR-191 increases breast cancer cell migration through TGFβ2 induction in a SMAD3-dependent manner (Summarized in Figure 7f).

### Anti-miR-191 therapy brings about reduction in MCF7 3D tumor spheroids volume

**Figure 8:**
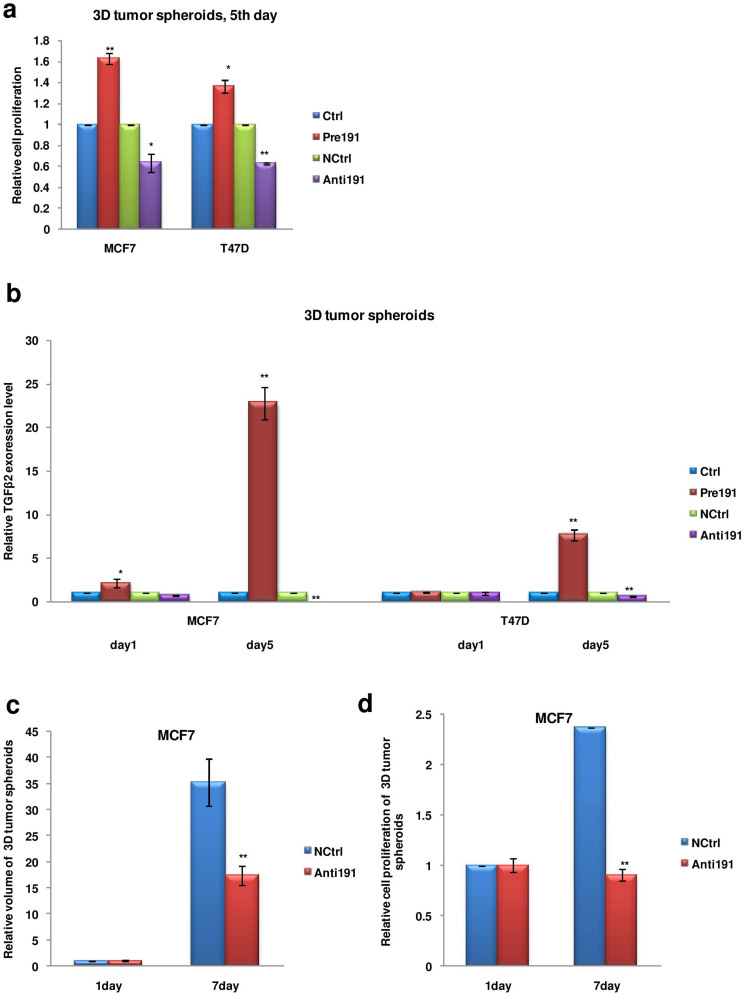
Effects of anti-miR-191 treatment in breast cancer cell 3D tumor spheroids. (a). MTT assay results showing difference in cell proliferation in 3D tumor spheroids generated from cells with differential level of miR-191. (b). qRT-PCR data showing TGFβ2 transcript level in the 3D tumor spheroids generated from cells with differential miR-191 levels. Significant induction in the level of transcripts was observed (at day 5/day 1) in the multicellular spheroids generated from cells with transient overexpression of miR-191. (c). Graph showing difference in volume of the tumor spheroids in response to anti-miR-191 treatment as compared to that of the control miRNA. (d). Graph showing results of MTT assay as a measure of cell proliferation of MCF7 spheroids in response to anti-miR-191 treatment. The graphical data points in a–d represent mean ± S.D of at least three independent experiments. (*P < 0.05, **P < 0.01). Error bars denote ± SD.

Recently, studies using 3D tumor models have gained considerable attention as they more realistically recapitulate the effect of endogenous tumoral microenvironment as compared to external exposure to hypoxic microenvironment in cells grown in 2D culture^51^,^52^. We generated 3D tumor spheroids for MCF7 and T47D cell lines transfected with miR-191 mimics or inhibitors and studied their growth characteristics. In agreement with the results in 2D culture conditions, spheroids generated with miR-191 overexpressed cells were found to be more proliferative while miR-191 inhibitor treated cells showed less proliferation (Figure 8a). A considerable induction in the level of TGFβ2 was also observed (at day 5/day 1) in the multicellular spheroids generated with cells treated with miR-191-mimic compared to that of treated with miR-191 inhibitor validating our results obtained using 2D culture (Figure 8b).

3D tumors develop an architecture that closely resembles *in vivo* solid tumors and are excellent tools for studying effects of anticancer drugs or treatments. MCF7 spheroids were generated and as a measure of the development of hypoxic core, *VEGFA* levels (a hypoxic marker) were measured. An increase in *VEGFA* levels were seen as spheroids grew from day 1 to day 7 showing development of hypoxic core (Supplementary data 7a). Since our studies establish miR-191 as an oncogenic miRNA in breast cancer, we wanted to see the effect of anti-miR-191 treatment on the 3D tumors. MCF7 spheroids were generated and anti-miR191/NCtrl treatment was given to the spheroids. Downregulation of miR-191 levels in the spheroids was confirmed by qRT-PCR (Supplementary data 7b). A single dose of anti-miR-191 therapy brought about a drastic reduction (~50%) in the 3D tumor volume at day 7 as compared to the control oligos (Figure 8c). This was accompanied by reduction in cell proliferation in anti-miR-191 treated cells suggesting anti-miR-191 therapy to be promising for breast cancer treatment (Figure 8d).

## Discussion

Solid tumors, such as breast carcinoma, are characterized by their heterogeneous nature, metastatic potential, regions of necrotic core and abnormal vasculature^53^. Hypoxic tumors are associated with resistance to chemotherapy and adjuvant therapies leading to poor prognosis^33^. Several HIF regulated genes and few miRNAs have been identified that play crucial role in hypoxia biology^5^,^8^. However, the mechanisms of hypoxia mediated effects still remain elusive calling for the need to identify novel mediators of hypoxia signaling. These candidates could serve to target notorious hypoxic tumors.

Here, we identify an oncogenic player in hypoxia biology that promotes breast cancer aggressiveness partly through induction of TGFβ signaling. Although recent studies by ours and Croce's group had implicated miR-191 as an onco-miR in breast cancer, its functional role in the hypoxic microenvironment has not been explored yet^26^,^28^. Here, we show that severe hypoxic microenvironment (0.2% pO_2_) often seen in solid tumors strongly induce miR-191 expression through dynamic binding of HIF-1α and HIF-2α to the HREs present upstream of miR-191. Although both the HIFs have been implicated in the induction of miR-191, the effect of HIF-1α seems to be more pronounced as evidenced by CHIP assay. It will be certainly interesting to see if miR-191 levels also correlate with the hypoxic quotient and markers in breast tumor patient samples.

Overall, miR-191 seems to be orchestrating the responses to hypoxia (summarized in Figure 7f). We show that miR-191 overexpressing cells show increased cellular proliferation, migration and therapy resistance as compared to the control cells under hypoxia and vice versa is seen when miR-191 is downregulated in hypoxic cells. Our previous work also shows cellular migration as part of functional domain of miR-191 that is partly mediated by downregulation of metastatic regulator SATB1 in hormone positive breast cancer^26^. Another study on aggressive breast cancer also confirmed the same^28^. In this study, we focused on the signaling activity of miR-191 under hypoxia and its effects on cell migration, which represents an essential component of the cancer metastasis cascade. In breast cancer, HIF and TGFβ signaling have been associated with increased cellular migration^54^,^55^. Our studies suggest that miR-191 may be a candidate for crosstalk between the two pathways leading to increased hypoxia induced breast cancer cell migration.

Active TGFβ-pathway is implicated in aggressive disease and poor prognostic outcome in breast cancer at the clinic^56^,^57^. The association of miR-191 with TGFβ-signaling has been suggested before by Elyakim's group in hepatocellular carcinoma based on gene expression profiling results^58^. A strong induction in TGFβ2 levels by direct miR-191 binding to its 3′UTR under hypoxia seems intriguing considering the fact that conventionally miRNAs are known as post transcriptional gene silencers^59^. Whether the high TGFβ2 levels seen in hypoxia are due to increased transcript stability or enhanced translation or both remain to be seen. Recent studies show that miRNA mediated upregulation of target transcripts may involve its interaction with the 5′UTR to facilitate polysome formation or alleviate translational repression, or binding to TATA box to enhance promoter activity or its cross-talk with various RNA binding proteins^15^,^16^,^17^,^18^,^60^,^61^. Since HuR, an RNA binding protein, is known to be associated with breast cancer, and its 3′UTR bears two miR-191 binding sites as well, we checked to see if miR-191 mediated regulation of HuR indirectly affects TGFβ2 levels^44^,^62^. Excitingly, we found the existence of an miR-191-HuR-TGFβ2 axis. We show that miR-191 directly targets HuR under hypoxia and mediates its downregulation. Since we also see that HuR downregulates TGFβ2 in hypoxia and that miR-191 mediated induction of TGFβ2 is reduced on HuR overexpression, thus, miR-191 targeting of HuR may be another mechanism to maintain high TGFβ2 levels under hypoxia. However, these observations though experimentally proven here raise two captivating questions: 1. Since HuR is conventionally shown to promote stability of target transcripts then how do we explain HuR mediated TGFβ2 downregulation? 2. Since, HuR has been correlated with high malignancy in various cancers then how do we explain miR-191 (oncomiR) mediated HuR downregulation^63^,^64^,^65^. To explain the first, we believe that transcript stability is highly dependent on the dynamic interactions of the protein and RNA. Thus, theoretically HuR levels can increase/decrease the level of mRNA depending on the binding and interaction between the two^17^,^66^. This may also involve its competition/cooperation with other miRNAs including miR-191 interacting with TGFβ2 transcript. Further, HuR was recently shown to suppress translation initiation of IGF-IR or promote destabilization of p16^INK4^ mRNA, thus expanding its repertoire from a stabilizer to repressor suggesting that in different context HuR may play different functions^67^,^68^. To explain the second question, several recent studies have shown that HuR downregulation may promote breast cancer aggressiveness. Like, downregulation of HuR has been shown to promote doxorubicin resistance in breast cancer cells^69^. Further, HuR levels were also found to be downregulated in breast cancer patients with bone metastasis^62^. Higher HuR levels led to good prognosis in metastatic breast cancer patients while lower HuR levels led to poor prognosis and overall survival in patients of metastatic breast cancer^62^. These studies suggest that miR-191 mediated doxorubicin resistance and increased migration in breast cancer cells may involve HuR downregulation as well. This study also brings to limelight a negative feedback loop that exists to regulate HuR levels under hypoxia. Thus, while hypoxia is known to induce HuR levels, simultaneously through miR-191 induction it brings about its downregulation.

In summary, our study unravels the hypoxia/HIF/miR-191/TGFβ pathway that plays an important role in breast cancer biology. Investigation of both therapeutic and prognostic potential of miR-191 should be actively pursued. We made an effort to generate breast tumor spheroids since they better reflect the *in-vivo* behavior of tumor cells and also serve as advanced tools for drug/therapy screening. Notably, a single dose of anti-miR-191 treatment brought about a drastic reduction in spheroid volume accompanied by a decrease in cell proliferation. This lays scope for further studies using animal models for development of a detailed protocol for using anti-miR-191 therapy for breast cancer treatment.

Lately, TGFβ-pathway is being assessed as an attractive therapeutic target for cancer treatment. One approach involves targeting TGFβ levels which are known to increase during cancer progression and correlated with poor prognosis. An antisense oligonucleotide drug API12009 (Trabedersen, Antisense, Pharma) has been developed to target TGFβ2 levels that has successfully passed phase I/II clinical trials^70^. Since our results from 3D spheroids suggest that miR-191 is a major regulator of TGFβ2 levels, thus, targeting miR-191 in tumors may have an added advantage during cancer treatment.

## Methods

### Cell culture

The human MCF7 & MM231, breast adenocarcinoma cell lines were a kind gift from Dr. Mircea Ivan (Indianapolis University, source ATCC). T47D breast cancer cell line was obtained from national cell repository at National Centre for Cell Science (NCCS), Pune, India. All the three types of cells were maintained in RPMI 1640 (GIBCO) medium supplemented with 100 U/ml penicillin, 100 ug/ml streptomycin and 10% fetal bovine serum. Cells were grown at 37°C in 5% CO_2_ incubator (Shell labs).

### Hypoxia/Stress/Radiation/Drug Treatment

For hypoxic stress, the cells were placed in hypoxia chamber (Invivo200, Ruskin, UK) maintained at different pO_2_ conditions (ranging from 0.2–1% oxygen) and 5% CO_2_. The cells growing under normal conditions (21% oxygen) were taken as control. For radiation treatment, cells were exposed to gamma rays (2Gray) and maintained for/scored for cell proliferation 24 hrs after exposure. Unexposed cells were taken as control. For drug treatment, doxorubicin at a concentration of 2 uM was added to the cells and cells were allowed to grow in the drug added media for 24 hrs. Cells treated with equal concentration of PBS were treated as controls. Each of the experiment was repeated three times.

### Transient Transfection

Cells were transfected with oligos (pre191/anti191 with their respective controls Ctrl/NCtrl), siRNA, esiRNA or plasmids using Lipofectamine 2000 for various experiments according to manufacturer's instructions. The precursor miRNA oligos (Ambion) were used at 30 nM, anti miRNA oligos (Ambion) at 60 nM, siRNA (Sigma) at 100 nM and esiRNA (Sigma) at 2 ug/ul final concentration. Each experiment was repeated three times in triplicates each.

### RNA isolation and Quantitative Reverse Transcription-Polymerase Chain Reaction (qRT-PCR)

Total RNA isolation from cell lines was done using Trizol reagent (Invitrogen)/RNA Isolation kit (GeneJET RNA purification kit, Thermo Scientific), according to manufacturer's instructions. The RNA was then reverse transcribed using Revertaid first strand cDNA synthesis kit (Fermentas) with oligo-dT as primer. cDNA formed was then further amplified for the predicted genes with respective primers using Power SyBr Green master mix kit (Ambion). GAPDH (Glyceraldehyde 3-phosphate dehydrogenase) was used as control for the normalization of the data. The sequence of primers used is given in Supplementary data 1.

### Stem-loop RT-PCR

Stem-loop RT-PCR was done to determine the level of miR-191 and RNU6B (used for normalization) in all the samples. cDNA was made with 500 ng of total RNA using specific RT primers as mentioned in Supplementary data 1. qPCR was performed using specific forward (miR-191F-, U6F-) and a common Stem-loop reverse universal primer (Reverse Primer) for both miR-191 and U6. The sequence of primers used is given in Supplementary data 1.

### Cellular assays

The cellular assays [MTT (3-(4,5-Dimethylthiazol-2-yl)-2,5-Diphenyltetrazolium Bromide) assay for cell proliferation, trypan blue assay for cell survival, Boyden chamber assay for migration; soft agar assay] were performed as previously described except that the transfected cells were exposed to hypoxia (0.2% pO_2_) for various time periods as indicated in the respective figures^26^. For invasion assay matrigel coated inserts were used. The cells were transfected with pre-191 along with esiTGFβ2/esiCtrl, or anti-191/NCtrl along with TGFβ2 treatment. 24 hrs post transfection the cells were transferred to the upper chamber of the transwell for migration/invasion and migration/invasion was allowed to take place in hypoxic conditons. Migrating cells were fixed with formalin (10 min at 37°C) followed by staining with crystal violet dye (0.1% for 2–3 min at RT). The stained membrane was then washed with water and quantification was done post elution with 10% acetic acid (595 nm, iMark microplate reader (BioRad). Invasion was quantitated after normalizing it with migration value.

For wound healing assay, the cells were transfected with miR-191 mimic/inhibitor/controls and 24 hrs post transfection scratches were made in the plate using 10 ul sterile tip (any cellular debris was removed by washing with PBS). The plate was then incubated in hypoxia chamber (0.2% pO2) in medium containing 10% FBS for 24 hrs and photographed under a light microscope (4× magnification). The area of migration was then estimated by choosing an arbitrary unit scale. The area of gap was then imaged and the distance covered was quantified by comparing the gap after hypoxic exposure to that of before putting the plate in hypoxic chamber.

### 3-D spheroid generation

The breast cancer 3-D spheroids were generated using MCF7 & T47D cells that were transiently trasfected with pre191/anti191/control oligos. Equal numbers of cells/well were plated on round bottom, 96-well plates coated with 50 ug/ml poly-HEMA [Poly (2-hydroxyethyl methacrylate), to prevent attachment and facilitate sphere formation], as mentioned earlier^71^. Compact multicellular spheroids with smooth margins were selected and pooled to perform various experiments at different time points. To study effect of anti-miR-191 treatment on MCF7 3D tumor spheroids, first, the spheroids were generated in the manner described above. Spheroids generated (24 hrs post seeding), were then transfected with anti-miR-191/NCtrl (60 nM) and change in size/morphology was imaged and quantified using MTT assay and scored for levels of miR-191 by using qRT-PCR after 7 days post-transfection. The experiment was repeated twice.

### Cell Cycle analysis

Cells were arrested in G0/G1 phase by treating them with serum starvation for 24 hours. After washings with complete media the arrested cells were transiently transfected (pre191/anti191/ctrls) and exposed to hypoxic microenvironment (pO_2_ 0.2%). Cells were fixed, stained (PI staining) and then analysed for cell cycle by FACS (BD Bioscience). The data obtained was analysed using Cyflogic software. The experiment was repeated three times.

### Mitochondrial Transmembrane Potential (ΔΨm)

Changes in mitochondrial membrane potential were analyzed using a mitochondria-selective dye (JC-1, Calbiochem). Cells were transiently transfected with miR-191 mimics (pre-191) or antisense (anti-miR-191) or respective control oligos (Ctrl and NCtrl) and exposed to hypoxic microenvironment (0.2% oxygen) for 24 hours. The cells were stained with JC-1 for 15 minutes at 37°C and then washed twice with PBS. Finally, the cells were resuspended in 500 ul PBS and green (excitation/emission, 485/530 nm)/red (excitation/emission, 550/595 nm) fluorescence was detected using a spectrofluorometer. The ratio of red to green fluorescence intensity was determined for each sample as a measure of ΔΨm. The experiment was repeated three times.

### Construction of luciferase reporter constructs

The predicted miR-191 binding site in the 3′UTR of HuR (positions: 4027–4033 and 4071–4077) and TGFβ2 (position: 5756–5784) was amplified and cloned in pMIR-REPORT™ vector to generate wild type 3′UTR-luciferase constructs. The miR-191 binding site in the 3′UTR of the HuR and TGFβ2 gene was mutated through site-directed mutagenesis with primers spanning the miR-191 binding site with indicated mutations using inverse PCR and luciferase activity was measured. The sequences of primers used for cloning and site-directed mutagenesis are mentioned in Supplementary data 1.

For promoter analysis, miR-191 promoter fragments predicted to encompass hypoxia response elements (HREs) were amplified. HREs (H2 & H3) encompassing HRE-consensus (ACGTG) were cloned upstream of a luciferase promoter vector PGL3-tk-luciferase (Promega) using Pfu DNA polymerase. All the clones were confirmed through PCR & restriction digestion. The sequence of the primers used for cloning of binding site and thus formation of reporter constructs along with the annealing temperature is mentioned in Supplementary data 1.

### Dual Luciferase assay

For luciferase assays, cells were cotransfected with 3′UTR luciferase constructs (above) along with pre191/anti191/ctrl oligos using Lipofectamine 2000 (Invitrogen) and exposed to hypoxic microenvironment for 24 hrs. pRL-TK was transfected in all the wells for normalization of transfection efficiency.

For confirmation of hypoxic microenvironment, EPO-HRE luciferase construct (along with pRL-TK) was transfected using Lipofectamine 2000 (Invitrogen) and 24 hrs post transfection cells were transferred to hypoxic chamber (0.2% pO_2_). 48 hrs post transfection cells were scored for luciferase activity and the luciferase activity of the cells kept at normoxia (21% pO_2_) were considered as control.

For effect of miR-191 on the HRE constructs (EPO-HRE, H2-HRE, H3-HRE and control vector pGL3-TK-Luc) were cotransfected with HIF1α/shHIF1α or their respective controls. 24 hrs post transfection cells were transferred to hypoxic chamber (0.2% pO_2_) and scored for luciferase activity 48 hrs post transfection.

The activities of Firefly (Photinus pyralis) and Renilla (Renilla reniformis) luciferase were quantified with the Dual Luciferase Reporter Assay (Promega) 48 hours post-transfection. The ratio of luciferase activity of Firefly to Renilla luciferase was taken as a measure of normalized luciferase activity. The experiments were performed thrice in triplicates each.

### Immunofluorescence

MCF-7 cells were grown as subconfluent monolayer cultures on polylysine (Sigma) coated glass coverslips. Cells grown on coverslips were then transfected with differential level of miR-191 (pre191/anti-191 and their respective controls) oligos and 24 hrs post –transfection cells were exposed to hypoxic microenvironment (pO_2_-0.2% for 24 hrs). Cells were washed, fixed and stained with primary antibody (TGFβ2; 1:50, Santa Cruz Biotechnology). Indirect immunofluorescence was done through anti-rabbit antibody (Alexa Fluor 488; 1:100, Invitrogen). Cells were imaged using Nikon fluorescent microscope (20×). The fluorescent intensity was then quantified using ImageJ software.

### Western Blotting

Cells were transfected with differential level of miR-191 (pre191/anti-191 and their respective controls) and exposed to hypoxic microenvironment. 48-hrs post-transfection, cells were lysed and equal amount of protein lysates were separated with 10% SDS-PAGE and transferred to nitrocellulose membrane. The membrane was then probed with a specific primary antibody at a dilution of 1:1000 for TGFβ2/HuR/HIF-1α (Santa Cruz Biotechnology) or 1:3000 for β-actin (Invitrogen) followed by washing and incubation with respective secondary antibody (anti-rabbit, HRP (horseradish peroxidase)-linked for TGFβ2 & HIF-1α, and anti-mouse HRP-linked for HuR & β-actin). The specific protein band was visualized by autoradiography using an enhanced chemiluminescence (ECL) kit (Amersham ECL Prime). For band densitometery, ImageJ software was used and the protein concentration was quantified.

### Chromatin Immunoprecipitation

To show HIF specific regulation of miR-191, CHIP assay was performed on MCF7 cells transfected with HA-tagged-HIF 1α/HIF 2α/parent vector and exposed to hypoxia for 24 hrs. To show endogenous HIF1α binding to miR-191 promoter in hypoxia, cells were exposed to normoxic or hypoxic microenvironment for 24 hrs. The cells were crosslinked with formaldehyde for 10 min at 37°C, washed with PBS (3–4 times) and resuspended in the RIPA buffer. Sonication was then performed to obtain DNA fragments of 100–500 bp in size. The fragment size was confirmed by gel electrophoresis on a 2% agarose gel. For chromatin immunoprecipitation, specific antibodies (1 ug each) against HIF 1α or HA tag (Santa Cruz Biotechnology) was added to the chromatin extract along with salmon sperm DNA (Sigma) and protein A-sepharose beads (30 ul) and incubated overnight. The beads were washed and eluted with 0.5%w/v SDS solution. Decrosslinking was done at 65° for 4–5 hrs and DNA was then purified with PCR purification kit (HiMedia). Rabbit IgG (1 ug) was used as control antibody while the chromatin extract without any antibody/beads treatment was used as positive control (input). For DNA sequence specific quantification qPCR was done with equal amount of chromatin extract using sequence specific primers. Ctrl primers amplify region lacking in HIF-binding sites. A list of primer sequences used for amplification is given in Supplementary data 1. The experiment was repeated thrice.

## Author Contributions

R.K. conceived the project and supervised the whole study. N.N. performed all the experiments. H.A. helped with western blot experiments. S.C. and S.G. helped with the spheroid studies. D.S. helped with the target predictions. R.K. and N.N. wrote the manuscript.

## Supplementary Material

Supplementary Information Supplementary Information
