# Supplementary material for: HIF-inducible miR-191 promotes migration in breast cancer through complex regulation of TGFβ-signaling in hypoxic microenvironment
Source: Sci Rep. 2015 Apr 13;5:9650. doi: 10.1038/srep09650 (PMC4394754; doi:10.1038/srep09650)

HIF-inducible miR-191 promotes migration in breast cancer through complex regulation of TGFβ-signaling in hypoxic microenvironment.

Neha Nagpal[1], Hafiz M Ahmad[2], Shibu Chameettachal[3], Durai Sundar[1], Sourabh Ghosh[3], Ritu Kulshreshtha[1]*

Supplementary data 1.- List of primers used

Supplementary data 2.- Functional assay for confirmation of hypoxic microenvironment or HIF activity
a. All the experiments have been performed by exposing the cells to 0.2% hypoxia (0.2% pO_2) in the hypoxic chamber for 24 hrs. The hypoxic microenvironment was confirmed by exposing the cells transfected with EPO-HRE to hypoxic microenvironment. Significantly high luciferase activity was observed in the cells exposed to hypoxic microenvironment. **b.** Activity of the HIF-1/2-α overexpression constructs or HIF-1/2-α specific shRNAs was confirmed through dual luciferase assay by cotransfecting EPO-HRE reporter plasmid in MCF7 cells. Graph represents relative luciferase activity. **c.** MCF7 cells were transfected with HIF-1α overexpressing plasmid/vector control (PC) and shHIF-1α/shCtrl to overexpress/ inhibit the expression of HIF-1α and western blotting was done to measure the HIF-1α levels. The graphical data points in a & b represent mean ± S.D of at least three independent experiments. (*P<0.05, **P<0.01). Error bars denote ± SD.

Supplementary data 3.- Confirmation of overexpression/ inhibition of specific molecules achieved by transient transfection
a,b. Level of miR-191 was transiently up/ downregulated by transfecting with pre-191 (Ambion) & anti-191 oligos (Exiqon) and exposed to hypoxic microenvironment for 24 hrs. The corresponding effect on the expression of miR-191 was then checked by qRT-PCR compared to that of their respective controls in MCF-7 cells. **c-e.** MCF-7 cells were transfected with pcdnA3.1-HuR or with esiRNA for HuR inhibition and exposed to hypoxic microenvironment for 24 hrs. The graph shows relative expression levels as determined by qRT-PCR (**c,d**) and western blotting (**e**) of HuR compared to that of respective control transfected cells. **f-h.** MCF-7 cells were transfected with TGFβ2 esiRNA or treated with recombinant TGFβ2 and the efficiency of esiRNA mediated inhibition or overexpression was confirmed through measuring TGFβ2 levels through qRT-PCR (**f,g**) or luciferase activity of pTPlux reporter construct (**h**). **i.** qRT-PCR data showing effect of ctrl or SMAD3 siRNA on SMAD3 levels in MCF7 cells. The graphical data points represent mean ± S.D of at least three independent experiments. (**P<0.01). Error bars denote ± SD.

Supplementary data 4.- Effect of miR-191 on TGFβ2 & HuR levels in the presence/absence of hypoxic microenvironment
a,b. Effect of miR-191 on TGFβ2 and HuR at protein levels in hypoxic microenvironment. The MCF7 cells with differential level of miR-191 were exposed to hypoxic microenvironment and analyzed for expression of TGFβ2 (**a**) and HuR (**b**) protein by western blotting and the relative protein levels were then quantified using imagej software for band densitometery. **c.** Level of miR-191 was differentially modulated and cells (MCF7) were exposed to hypoxic microenvironment, Immunofluorescence was done by using an antibody specific for TGFβ2 to compare the difference in fluorescence intensity observed. **d.** Cells were transfected with esictrl or esiHuR oligos in normoxia and qRT-PCR was performed. The results show that under normoxic conditions HuR is unable to downregulate TGFβ2 levels in breast cancer cell lines. **e-g** qRT-PCR was done to find out the effect of miR-191 on TGFβ2 & HuR levels in both normoxic and hypoxic microenvironment in a panel of breast cancer cell lines MCF7 (**e**), T47D (**f**) & MM231 (**g**). We found that the effect was minimal in the absence of hypoxic microenvironment. The graphical data points in a-g represent mean ± S.D of at least three independent experiments. (*P<0.05, **P<0.01, *^P>0.05<0.1). Error bars denote ± SD.

Supplementary data 5.- HuR is a direct target of miR-191.

a. Diagram showing sequence and position of miR-191 binding sites (wild type: HuR B1 & B2, mutant type HuR Mut B1) in HuR 3'UTR. **b,c.** 3'UTR luciferase activity of lucifease constructs (individually cloned: HuR B1, HuR B2, Both together: HuR B1&2) in response to differential miR-191 levels. It was found that miR-191 overexpression led to decrease in luciferase activity with HuR B1 and HuR B1&2 constructs (**b**) while lesser effect was observed with HuR B2 (**b**) or mutated HuR B1 sites (**c**). Therefore, miR-191 mediated downregulation of HuR is mediated mainly through binding to the HuR B1 site present in the HuR 3'UTR. The graphical data points in b & c represent mean $\pm$ S.D of at least three independent experiments. (*P<0.05, **P<0.01, *^P>0.05<0.1). Error bars denote $\pm$ SD.

Supplementary data 6.- Diagram showing putative miR-191 and HuR binding sites in TGFβ2 3'UTR

Diagram showing in-silico analysis data of the 3'UTR region of TGFβ2 for the putative HuR consensus sequences and miR-191 binding sites using RNAhybrid, ARE score and catRAPID softwares. The 3'UTR region containing the miR-191 and HuR binding sites of TGFβ2-3'UTR was cloned and checked for luciferase activity. Primer details are given in **Supplementary data 1**.

Supplementary data 7.- Validation of hypoxic microenvironment and transfection efficiency in the tumor spheroids

a. To recapitulate the endogenous hypoxic microenvironment 3D tumor spheroids were manifested and the level of *VEGFA* was sought as a marker of hypoxic microenvironment. **b.** The 3D tumor spheroids of MCF7 were transfected with anti-miR91/NCtrl and the effectiveness of transfection was confirmed by qRT-PCR. The graph shows miR-191 levels in antimiR-191/NCtrl treated MCF7 spheroids on day 1 and 7. The readings were normalized using U6. . The graphical data points in a and b represent mean $\pm$ S.D of at least three independent experiments. (*P<0.05, **P<0.01). Error bars denote $\pm$ SD.

Supplementary Data 1- List of Primers

Primer Detail	Primer Sequence	Annealing Temperature
**miR-191 RT primer**	GTCGTATCCAGTGCAGGGTCCGAGGTATTCGCACTGGATACGACCAGCTG	42^0C
**RNU6B RT primer**	GTCGTATCCAGTGCAGGGTCCGAGGTATTCGCACTGGATACGACAAAATATGGAAC	42^0C
**miR-191 forward primer**	CGCGCAACGGAATCCCA	60^0C
**RNU6B forward primer**	GCCCCTGCGCAAGGATGAC	60^0C
**Stem-loop reverse universal**	GTGCAGGGTCCGAGGT	60^0C

Primers designed to detect transcript levels of various shortlisted target genes

Primer Detail	Forward Primer	Reverse Primer	Annealing Temperature
**PTGS2**	TCAGTAGGTGCATTGGAATCAAGC	TAGCAGTCCTGAGCTGAGGTTTACC	60^0C
**BMP4**	AGCATGTCAGGATTAGCCGATCG	AGTCATTCCAGCCCACATCGC	60^0C
**SMAD3**	GAGATGATGGGCTAAACAGGCAAC	CACTGTCCTTGGGAGGAGAGACC	60^0C
**TGFβ2**	AGTCATACCACCTTTCCGATTGCC	AAAGCATGGTGCAAACATCTCTCTG	60^0C
**TGFβ1**	TCCTGGCGATACCTCAGCAAC	TGAACCCGTTGATGTCCACTTG	60^0C
**JUN**	GTGTGCACGAGTGGGAAGG	GATCGAATGTTAGGTCCATGGAG	58^0C
**FOS**	AATGACCCTGAGCCCAAGCC	AGCTCTGTGGCCATGGCC	58^0C
**CTGF**	AGCGTGCTCACTGACCTGCC	TCACTTGCCACAAGCTGTCCAG	60^0C
**VEGFA**	GCTACTGCCATCCAATCGAGAC	CTATGTGCTGGCCTTGGTGAG	58^0C
**HuR**	ACCAATGTGAAAGTGATCC	TATGAATTCTTATTTGTGGGACTTGTTG	55^0C

Primers designed to amplify HIF binding sites in miR-191 promoter for CHIP assay			
**Ctrl**	AGGCGCGCTTCGATGACG	AGGCAGGCGAGGGCATGG	60^0C
**H1**	GCTCCCTGTGCCATGTTGTCC	TCACCATGTTGGCCAGGCTG	60^0C
**H2**	AGCCTGGCCAACATGGTGAAAC	TGCCTCCTTCAGTGTGATGCC	60^0C
**H3**	ACTAACTGCACGGTGACTCCTGC	ATCAGGACTGCAGCTTGGCTG	60^0C
**H4**	CATCTGACTCTGGCTCCCAAGG	AGGGTTCACCATGTTGGCCAG	60^0C
**H5**	ACGACAAATCCACGCAGCCTC	TGATAAACGGAAACCGCGTGC	60^0C
**H6**	TGTTCTGTGGCCCAGGTGAGC	AGCTGCTTTTGGGATTCCGTTG	60^0C
**Primers designed to clone the 3'UTR luciferase reporter constructs**			
**TGFβ2**	ATTGAGCTCTGCTTTGGCTTT CTGGTTCTATG	ATTAAGCTTGAGGAGTCTGGTCTT GTAGGTAGC	57^0C
**Mut-TGFβ2**	CAGATATAACAAGAGCCACG TGCTTTCTCCCCTTGGTTGTT TGGGATCAGCTACTTGC	GCAAGTAGCTGATCCCAAACAAC CAAGGGGAGAAAGCACGTGGCTC TTGTTATATCTG	57^0C
**HuR B1**	ATAACTAGTGGCAATTGGCG TGTAATGATG	ATTAAGCTTCACATGGTCATGGTC AAAGAGG	57^0C
**HuR B2**	ATAACTAGTCACCTCTTTGAC CATGACCATGTG	ATTAAGCTTTGGGAATCGGTTAAG AGAGCCTC	57^0C
**HuR B1 &2**	ATAACTAGTGGCAATTGGCG TGTAATGATG	ATTAAGCTTTGGGAATCGGTTAAG AGAGCCTC	60^0C
**Mut-HuR B1**	TCCACCAGAAGAGAAGCCTT TTGGCTTGGTTTGGGGCCACC TCTTTGAC	GTCAAAGAGGTGGCCCCAAACCA AGCCAAAAGGCTTCTCTTCTGGTG GA	66^0C
**H2-HRE**	ATAACGCGTAGCCTGGCCAA CATGGTGAAAC	ATACTCGAGAGAGTGCTTGGTTCC AAGGTATGTG	60^0C
**H3-HRE**	ATACTCGAGGCATGAGGTAT GGCAGAGG	ATAACGCGTACCACTGCCCTTATC TTGCCTG	60^0C

Supplementary Data 2

a

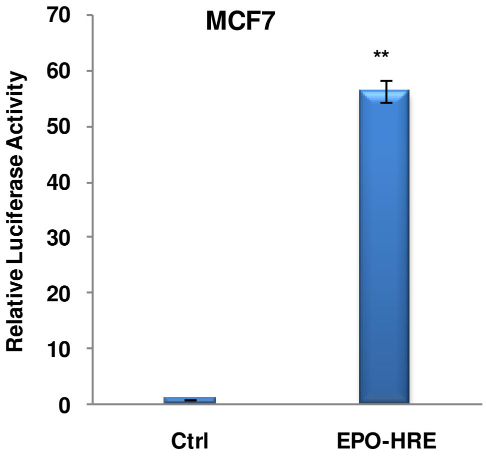

b

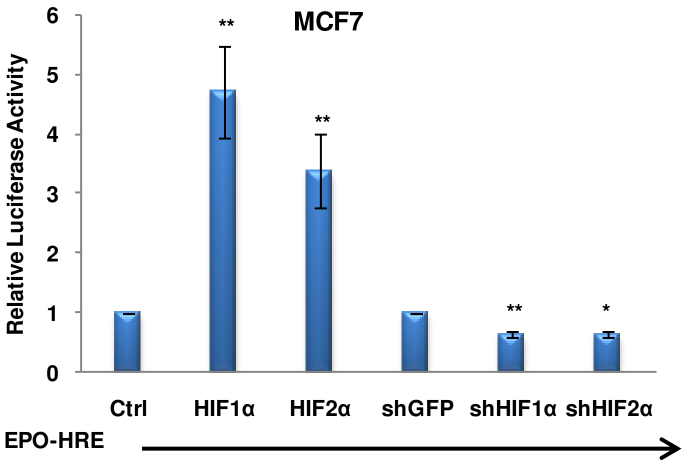

c

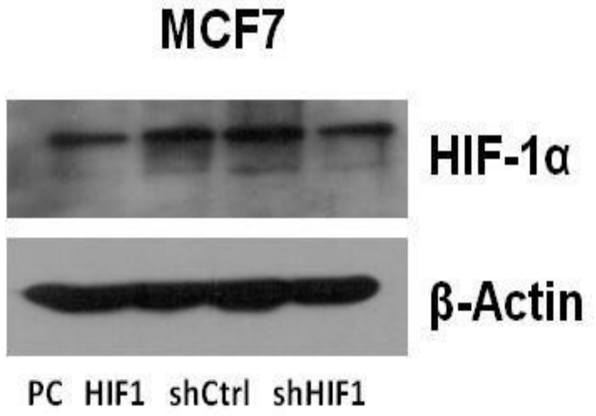

Supplementary Data 3

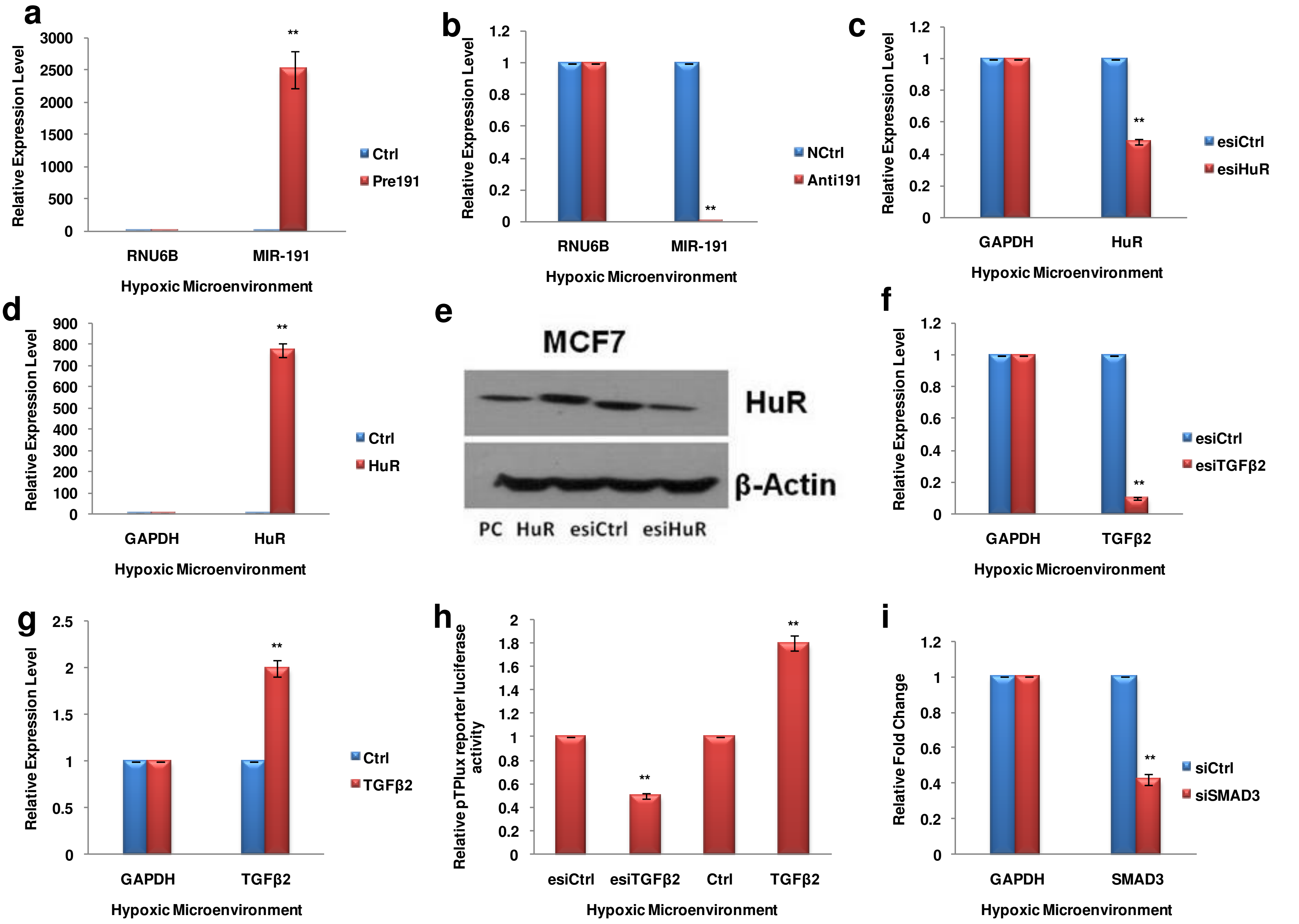

Supplementary Data 4

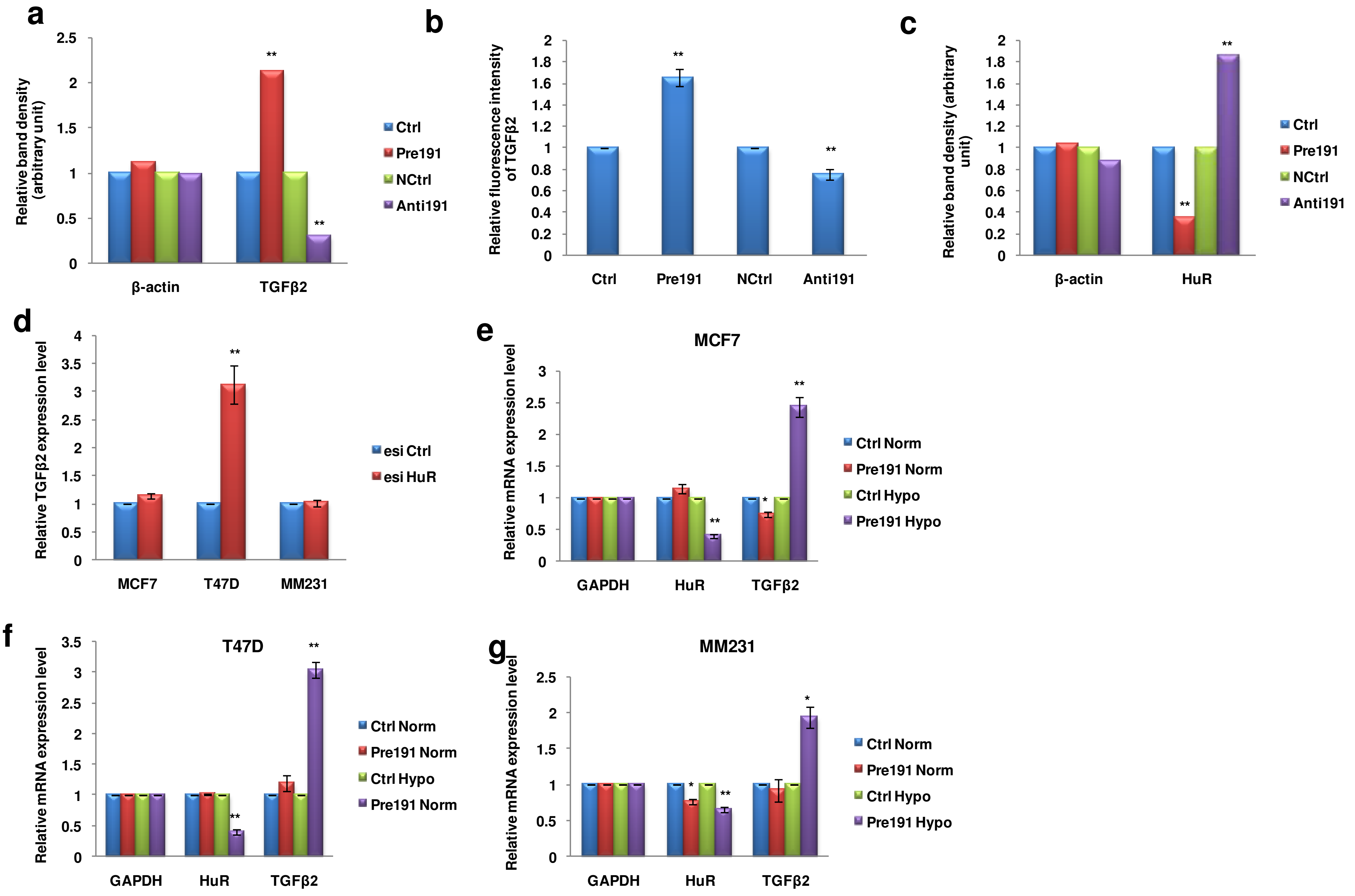

Supplementary Data 5

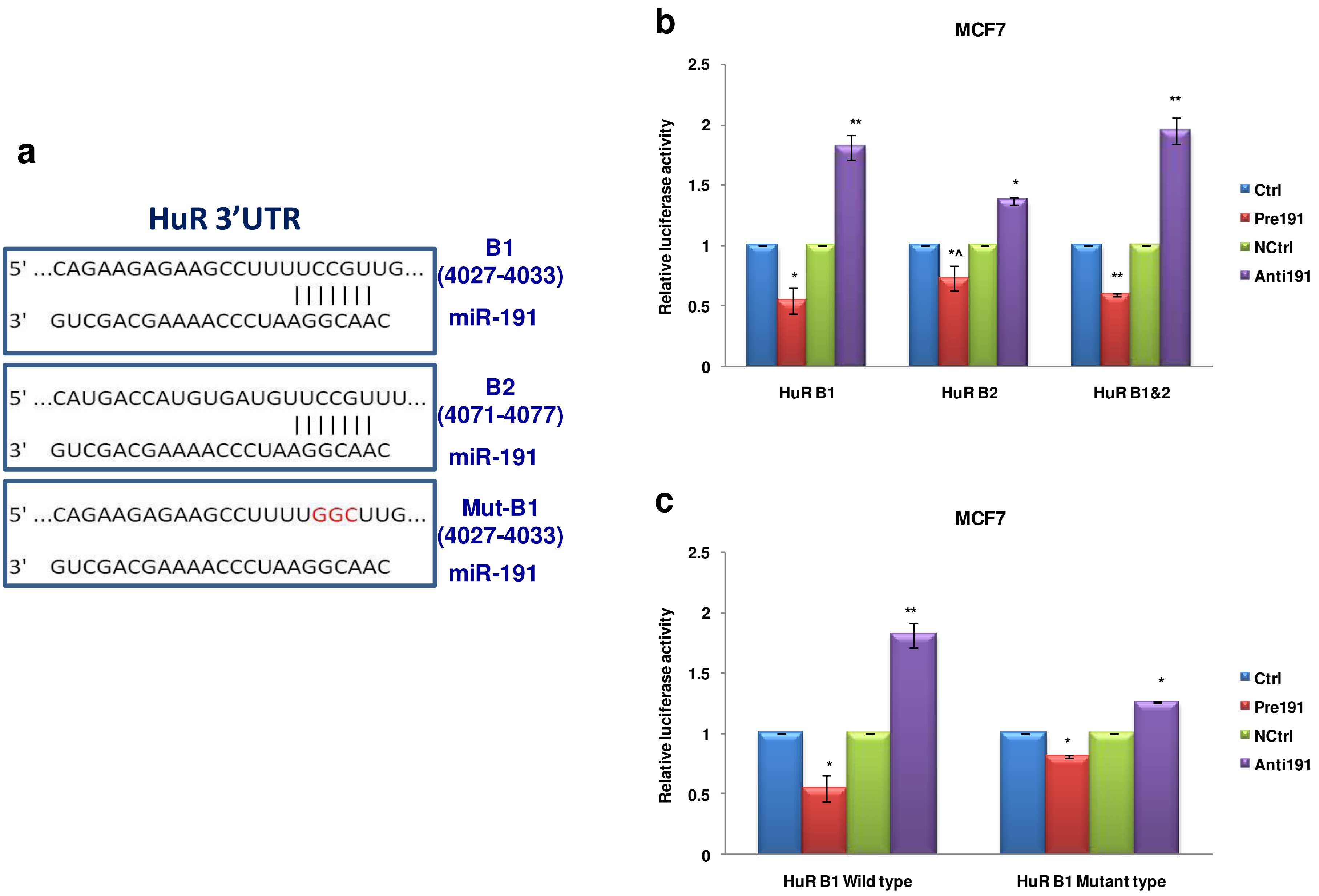

Supplementary Data 6

TGFβ2

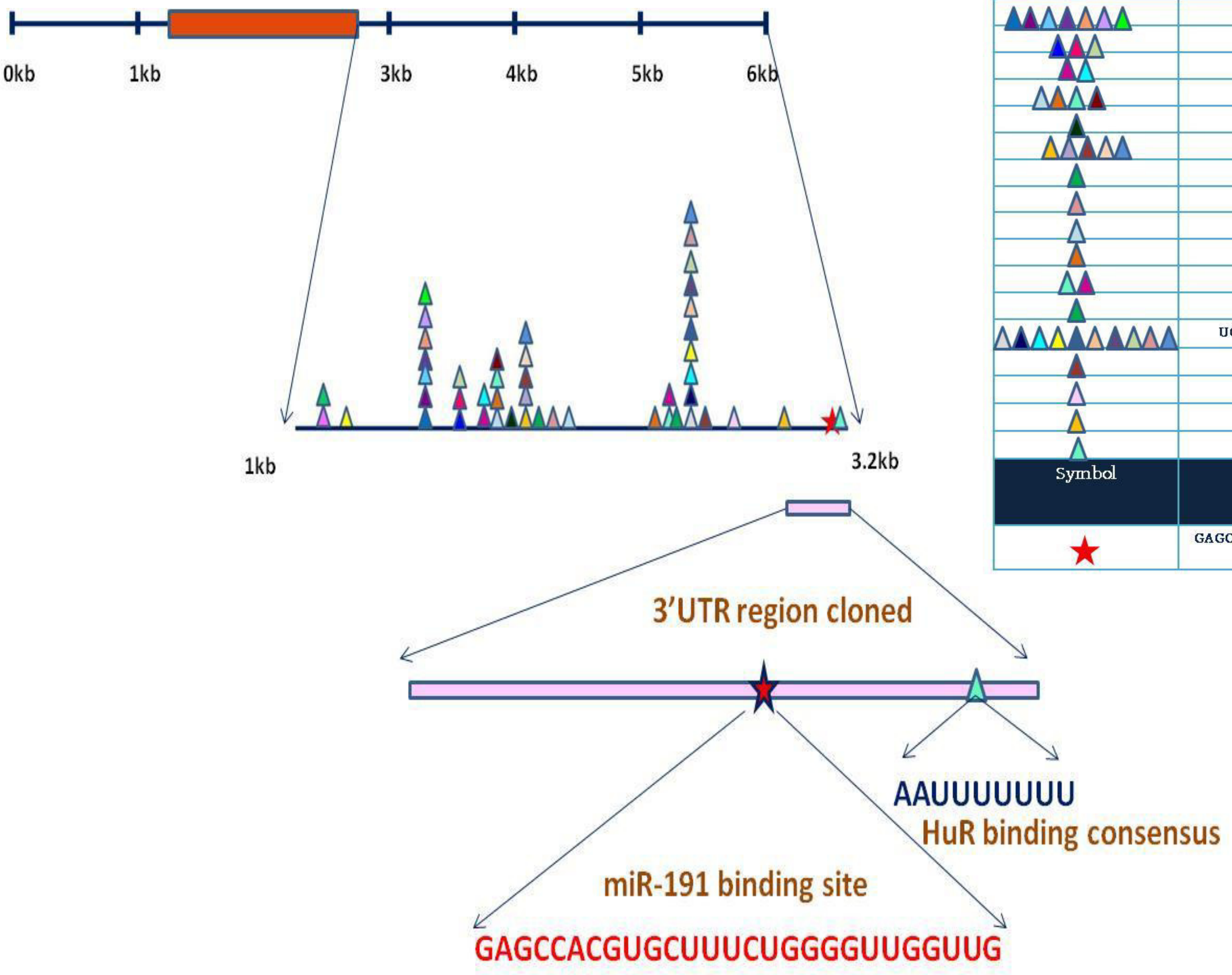

Symbol	HuR binding consensus	Position in TGFβ2-3'UTR
▲▲	AAUUUUUUUU	256-267
▲	ACUUGAUUU	400-408
▲▲▲▲▲▲▲	AGUUUUUUUUUUUCUUUU	785-795
▲▲▲	UUUUCCUUUGUUUU	920-933
▲▲	UGUUUCUUUU	1158-1167
▲▲▲▲	CAUUUUUUUUUU	1222-1233
▲	UGUUUCUUU	1261-1269
▲▲▲▲▲	CUUUUCUUUUUUUUGUUU	1305-1322
▲	UUUUCAUUU	1456-1464
▲	CCUUGGUUU	1529-1537
▲	UUUUUCUUU	1598-1606
▲	CUUUUAUUU	2225-2233
▲▲	AUUUUGUUUU	2265-2274
▲	UAUUAUUUU	2276-2284
▲▲▲▲▲▲▲▲▲▲	UGUUUUUUUUUUUUUUAAUUU	2346-2366
▲	UCUUUAUUU	2382-2390
▲	AAUUAUUUU	2469-2477
▲	AUUUAGUUU	2883-2891
▲	AAUUUUUUU	3163-3171
**Symbol**	**miR-191 Binding Site**	**Position in TGFβ2-3'UTR**
★	GAGCCACGUGCUUUCUGGGGUUGGUUG	3059-3085

Supplementary Data 7

a

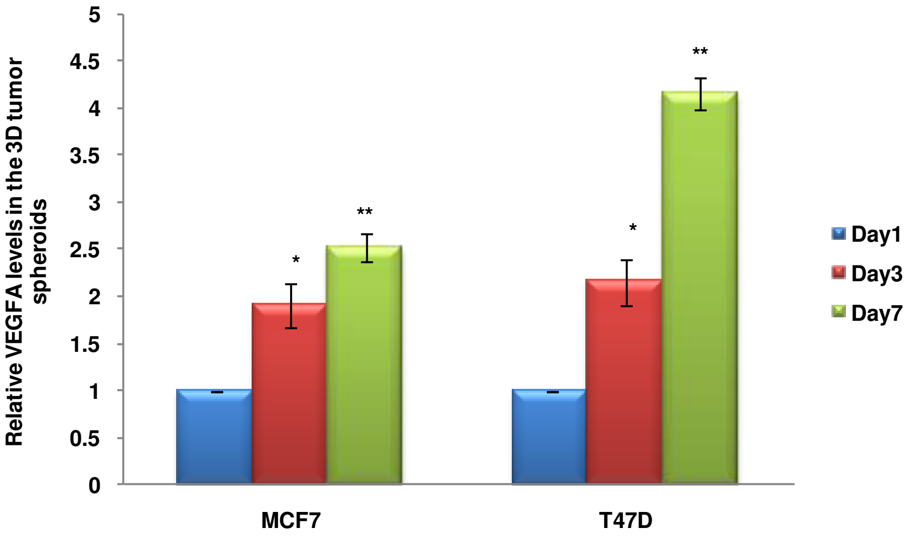

b

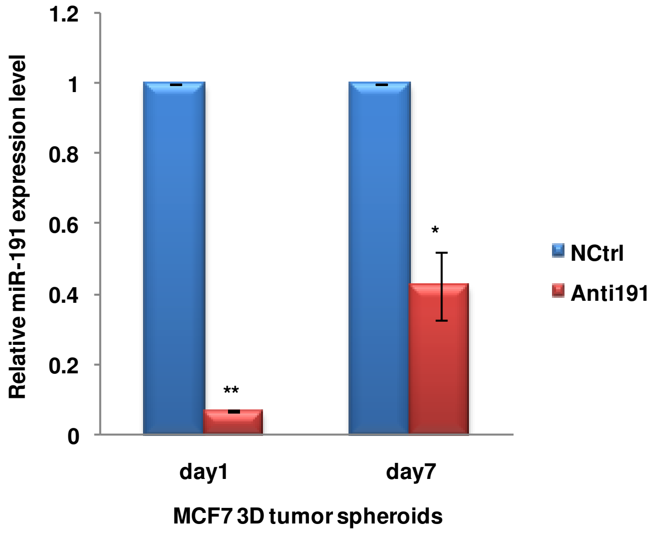

Supplement: Supplementary Information [file srep09650-s1.pdf]
